# The therapeutic potential of bone marrow mesenchymal stem cells in premature ovarian failure

**DOI:** 10.1186/s13287-018-1008-9

**Published:** 2018-10-04

**Authors:** Yantao He, Dongmei Chen, Lingling Yang, Qiaoni Hou, Huiming Ma, Xian Xu

**Affiliations:** 1 Key Laboratory of Fertility Preservation and Maintenance of Ministry of Education, Ningxia Medical University, No. 1160 Shengli Street, Yinchuan, 750004 Ningxia China; 2 The Center of Reproductive Medicine of General Hospital of Ningxia Medical University, Ningxia Medical University, No.804 Shengli Street, Yinchuan, 750004 Ningxia China; 3 Institute of human stem cells research of General Hospital of Ningxia Medical University, Ningxia Medical University, No. 804 Shengli Street, Yinchuan, 750004 Ningxia China

**Keywords:** Premature ovarian failure, Bone marrow mesenchymal stem cells, Transplantation

## Abstract

With the development of regenerative medicine, a variety of mesenchymal stem cells (MSCs) are increasingly considered for the treatment of premature ovarian failure (POF). Reportedly, bone marrow-derived MSCs (BMSCs) improve the ovarian reserve, which mainly depends on homing and paracrine activities. Furthermore, paracrine factors secreted by these stem cells play an important role in ovarian recovery. Relevant studies indicate that BMSC transplantation has some positive effects on the treatment of POF in animals, but BMSCs are not widely applied in clinical therapy. Clinical trials are ongoing despite the fact that several patients experiencing BMSC transplantation recover their normal menstrual cycles and even give birth to babies. In this review, we discuss the possible therapeutic mechanisms of BMSCs for POF, migration, antiapoptosis, antifibrosis, angiogenesis, anti-inflammation, immunoregulation, and oxidative stress, which provide the theoretical basis for further study and clinical therapy.

## Background

Due to the effects of various factors, especially the wide use of chemotherapy, there is an increasing trend for women suffering from premature ovarian failure (POF), leading to their infertility which is seriously upsetting for the patient. POF is a common gynecological endocrine disease that occurs in women under the age of 40 years and is characterized by amenorrhea, hypergonadotropinemia, and estrogen deficiency, affecting 0.9–1.2% of women [1] . The etiology of POF is unknown, but it is classified as genetic, autoimmune, and iatrogenic, and can present as idiopathic [2]. Estrogen supplementation remains the main treatment, which improves the symptoms of osteoporosis caused by the low estrogen levels to some extent [3]. However, thus far, there is no cure for POF. Of course, estrogen supplementation also increases the risk of cancer, such as mammary cancers and endometrium carcinoma. Recently, with the emergence of regenerative medicine, many studies using stem cell therapy for POF have been conducted. Given their pluripotency and low immunogenicity, bone marrow-derived mesenchymal stem cells (BMSCs) are believed to have therapeutic potential for POF. BMSCs play an important role in restoring injured ovaries in POF induced by cisplatin in rats [4]. Moreover, BMSCs also restore ovarian hormone production and reactivate folliculogenesis in a mouse model of POF caused by chemotherapy [5]. Other research suggests that BMSCs reduce granulosa cell apoptosis induced by cisplatin and perimenopause [6]. These studies show that BMSCs are effective in the treatment of POF models. Autologous BMSCs were applied for the clinical treatment of patients with idiopathic POF, and the results showed that two cases (20%) recovered menstruation at 3 months after transplantation and one of them (10%) became pregnant and delivered a healthy baby [7] . Another study showed that estrogen and anti-mullerian hormone (AMH) levels were rising in 86.7% of patients 1 month after autologous BMSC transplantation, and this change continued throughout the 48-week follow-up period. In addition, 18 patients (60%) started to ovulate, with ovum sizes ranging from 12 to 20 mm, which indicated that the autologous BMSCs may improve the conditions in patients with POF [8]. The therapeutic effects of autologous BMSCs in patients with POF are summarized in Table 1. There is no wide application of clinical therapy for patients with POF due to some of the limitations of BMSC transplantation and, thus, their clinical availability still requires further study.

**Table 1:** The therapeutic effects of autologous BMSCs on patients with premature ovarian failure [8]

Transplantation method	Isolated volume, ml	Transplantation numbers (million)	Patients, *n*	Hormone improvement, *n* (%)	Menstruation, *n* (%)	Spontaneous pregnancy, *n* (%)
Laparoscopy catheter	60	3–5	30	26 (86.7)	Unclear	1 (3.3)
Laparoscopy	10	Unclear	10	Unclear	2 (20)	1 (10)

Bone marrow-derived mesenchymal stem cells (BMSCs) were isolated from the iliac crest of the patients and were transplanted into the ovary by laparoscopy

## The present situation in POF

Women suffering from POF are severely affected both physically and mentally, and must face infertility, amenorrhea, osteoporosis, some cardiovascular diseases, and more. POF is mainly associated with low numbers of antral follicle and granulosa cell activities, which results in low estrogen levels in the serum. Presently, POF is mainly improved by hormone replacement therapy, which has some side effects. Therefore, clinicians are looking for new therapies for POF, and BMSC transplantation is a promising treatment.

## Characteristics of BMSCs

**Fig. 1:**
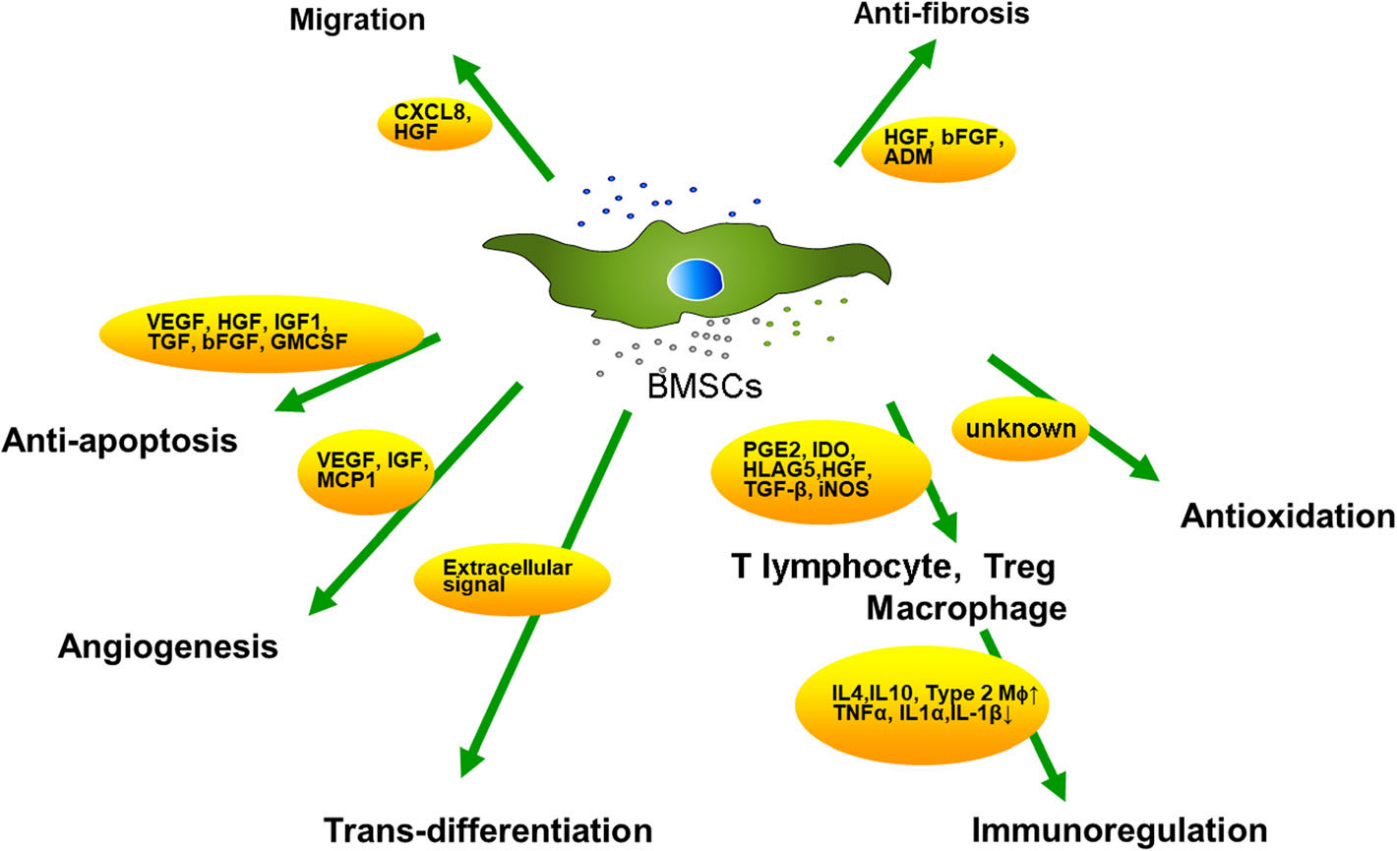
The possible mechanisms of bone marrow-derived mesenchymal stem cells (BMSCs). The migration of BMSCs is associated with CXCL8 and HGF. HGF, VEGF, IGF-1, TGF, bFGF, and GMCSF, secreted by BMSCs, contribute to inhibiting apoptosis. VEGF and HGF play an important role in angiogenesis. The mechanism of antioxidation is still unknown. ADM adrenomedullin, bFGF basic fibroblast growth factor, CXCL8 C-X-C chemokine ligand-8, GMCSF granulocyte macrophage colony-stimulating factor, HGF hepatocyte growth factor, HLAG5 human leukocyte antigen G5, IDO indoleamine 2,3-dioxygenase, IGF1 insulin-like growth factor-1, IL interleukin, iNOS inducible nitric oxide synthase, MCP1 monocyte chemoattractant protein 1, PGE2 prostaglandin E2, TGF transforming growth factor, TNF tumor necrosis factor, Treg regulatory T, VEGF vascular endothelial growth factor

BMSCs are a type of adult stem cell with a low immunogenicity. They are widely present in the bone marrow microenvironment and have the potential for renewing themselves and differentiating into many different tissue cells, such as bone, cartilage, adipocytes, and so on under certain conditions [9]. Furthermore, BMSCs are easy to isolate and amplify in vitro and, due to their paracrine and immunomodulation functions, they migrate to the site of injured tissue and also differentiate into specific cell types in the tissue under the induction of certain factors to reconstruct the local microenvironment. By enhancing the function of endogenous cells and regulating the immune response, they are involved in the repair of tissue damage, which makes BMSCs an ideal seed cell for transplantation. Despite the low survival rate and limited differentiation potential after BMSC transplantation, some encouraging results have been obtained. Autologous stem cell transplantation for the clinical treatment of POF is a great step [7, 8]. BMSCs improve the ovarian reserve of POF, and this is associated with the following aspects. BMSCs are induced by cytokines and migrate to the damaged tissue but do not differentiate into oocytes, according to the present study [10]. They secret certain cytokines that are helpful for antiapoptosis and antifibrosis, including vascular endothelial growth factor (VEGF), insulin-like growth factor (IGF), and hepatocyte growth factor (HGF), to help ovarian restoration. They also protect ovarian function by inhibiting the inflammatory response and decreasing oxidative stress. They regulate the immune system through certain cytokines, such as interleukin (IL)-6. These possible mechanisms are summarized in Fig. 1.

## Migration and homing of BMSCs

Simply put, the homing of stem cells means that they can directly and impulsively migrate to the injured tissue and survive there under the stimulation of multiple factors, which facilitates ovarian recovery. Liu et al. demonstrated that BMSCs home to the ovaries via the blood circulation to restore ovarian structure and function in POF model rats, and they found that the BMSCs mainly exist in the ovarian hilum and medulla and also in the cortex, but were not in the follicles or corpus lutea [4]. Another study also suggests that BMSCs localize and survive in the injured ovary after transplantation, thus promoting the ovarian recovery of histological structure and endocrine function [11]. Chemokine and growth factor receptors, such as the receptors for IL-8 (CXCL8) and HGF, located on the surface of BMSCs are involved in the migration and homing of BMSCs [12, 13]. MicroRNA-21 (miR-21) facilitates BMSC migration by upregulating matrix metalloproteinase (MMP)-2/MMP-9, potentially via the phosphatidylinositol-3-OH-kinase/protein kinase B (PI3K/Akt) pathway in vitro [14]. Another study found that stem cells migrate into the ovary and differentiate into a variety of cells, including theca cells, granulosa cells, corona radiata cells, and vascular endothelial cells, thus revealing that BMSCs might contribute to ovarian regeneration by enhancing angiogenesis and steroidogenesis [10] which is extremely controversial for differentiation. However, whether BMSCs differentiate into oocytes after migrating to injured tissue is still not known. It is widely accepted that the paracrine effect of BMSCs is the key rather than differentiation. Further studies are needed to explore whether BMSCs differentiate into ovarian cells, which would also be valuable for BMSC transplantation applied as a clinical therapy.

## Paracrine effects of BMSCs and conditioned medium

BMSCs secret chemokines, growth factors, hormones, and so on, to influence adjacent cells (the paracrine effect). Paracrine signaling is important in angiogenesis, anti-inflammation, immunoregulation, antiapoptosis, and antifibrosis, thus improving the microenvironment to promote the recovery of the damaged tissue. Kinnaird et al. suggested that BMSCs express genes relative to arteriogenic cytokines, such as VEGF, fibroblast growth factor-2 (FGF-2), and IL-6, and promote arteriogenesis by paracrine mechanisms in vitro and in vivo [15].

Given the paracrine effect of BMSCs, a study also used the conditioned medium from BMSCs, instead of BMSCs themselves, to examine the therapeutic effect on the damaged ovary, and the results showed that conditioned medium had a similar effect on the injured ovary [16], thus suggesting that perhaps conditioned medium from BMSCs in vitro, induced by the same factors in vivo, could also be therapeutic for the disease. Consequently, conditioned medium may be an effective therapy applied in the clinic, and even that artificial cytokines could be a reality someday. However, there is still a need for relevant studies on the effect of conditioned medium for POF.

## Antiapoptotic effects of BMSCs

Some studies show that BMSCs inhibit the apoptosis of granulosa cells in an animal model of POF [6], which is mainly associated with the antiapoptosis growth factors secreted by BMSCs. Fu et al. detected certain cytokines, including VEGF, HGF, and IGF-1, in the BMSC cultures and found that BMSCs inhibited the apoptosis of granulosa cells and upregulated B-cell lymphoma-2 (Bcl-2) in vivo [17]. Another study revealed the protective effect of VEGF in frozen-thawed granulosa cells by inhibiting apoptosis [18]. Uzumcu et al. found that HGF had an antiapoptotic effect on granulosa cells in vitro [19]. IGF-1 promotes granulosa cell proliferation to increase steroid hormone secretion, and aromatase (Cyp19) stimulation by follicle-stimulating hormone (FSH) in ovarian granulosa cells depends on the activation of the IGF-1 receptor-signaling pathway [20]. There is a study showing that BMSCs reverse the increased cyclin-dependent kinase inhibitor 1A (p21) and Bcl-2-associated X protein (bax), and decreased proto-oncogene (c-myc) mRNA expression managed by cisplatin in granulosa cells [6]. It is these growth factors that probably play an important role in the antiapoptosis of granulosa cells by downregulating p21 and bax and upregulating c-myc. miR-21 is a microRNA that is associated with apoptotic regulation and, thus, overexpression of miR-21 in BMSCs inhibit granulosa cell apoptosis in POF by targeting phosphatase and tensin homolog deleted on chromosome ten (PTEN) and programmed cell death 4 (PDCD4) [21]. The antiapoptotic effect is also associated with transforming growth factor (TGF), basic fibroblast growth factor (bFGF), and granulocyte macrophage colony-stimulating factor (GMCSF) [22].

## Antifibrotic effects of BMSCs

Fibroblasts proliferate excessively and deposit extracellular matrix in the ovary and, beyond a certain range, this can form ovarian fibrosis which is related to POF. Researchers observed ovarian atrophy and fibrosis in the morphology in animal models of POF, with exhausted functional follicles [23, 24]. Surprisingly, the collagen fiber content was obviously reduced after BMSC transplantation [24]. Ovarian fibrogenesis is associated with certain cytokines, including MMPs, tissue inhibitors of MMPs (TIMPs), TGF-β1, VEGF, and endothelin-1 (ET-1) [25]. The antifibrotic effect is associated with HGF, bFGF, and adrenomedullin (ADM) [22].

BMSCs inhibit the proliferation of fibroblasts and decrease the deposition of some extracellular matrix, thereby improving ovarian fibrosis. However, the antifibrotic molecular mechanism of BMSCs needs further study.

## Angiogenesis

Angiogenesis is also of importance in ovarian recovery; this provides nutrition for the injured ovary. Factors, such as VEGF, secreted by the BMSCs are associated with angiogenesis. Research reports that some factors managed by BMSCs are increased, including VEGF and FGF2 and especially angiogenin, which increases dramatically, thus stimulating neovascularization and facilitating blood perfusion of the grafts after cryopreserved ovarian cortex transplantation [26]. It is reported that BMSCs promote angiogenesis via the α6β1 integrin receptor [27]. A study shows that BMSCs differentiate into endotheliocytes and pericytes for angiogenesis after they are injected into uterine scar tissue in the rat [28]. Coculturing endothelial progenitor cells and BMSCs enhances their proliferation and angiogenesis through platelet-derived growth factor (PDGF) and translocation-associated (Notch) signaling [29]. In addition, BMSC-derived angiogenin has a positive effect on regulating angiogenesis in grafted human ovarian tissue [30]. Another study reports that BMSC transplantation, combined with the HGF gene, may have an obvious effect on angiogenesis compared with BMSC transplantation alone [31]. LIM-domain only 2 (LMO2), a key transcription factor for angiogenesis, plays an important role in angiogenesis via TGF-β1 and HGF [32]. HGF upregulation enhances angiogenesis in mice [33]. VEGF and HGF synergistically promote angiogenesis after islet transplantation [34]. VEGF promotes the length, area, and branch point number of the induced vessels, while HGF contributes to the vascular area growth. Moreover, the combination of VEGF and HGF leads to an increased vascular diameter [35]. MMPs serve a purpose in regulating capillary diameter and possibly in stabilizing the nascent vessels. BMSCs contribute to angiogenesis associated with membrane type 1 (MT1)-MMP [36]. Angiogenesis is involved in IGF and monocyte chemoattractant protein 1 (MCP1) [22].

## Anti-inflammatory effects and immunoregulatory effects of BMSCs

Anti-inflammation and immunoregulation may be other mechanisms by which BMSCs improve the injured ovary. Research reveals that cryopreserved BMSCs via intravenous administration help in experimental pelvic inflammatory fertility recovery [37]. Yin et al. revealed that ovarian function in POF mice was recovered by the regulation of regulatory T (Treg) cells and associated cytokines after human placenta-derived mesenchymal stem cell (hPMSC) transplantation [38]. It is reported that ovarian restoration in mice with POF is involved in Th17/Tc17 and Th17/Treg cell ratios through the PI3K/Akt signaling pathway, which shows that hPMSCs regulate the immune system [23]. Similarly, human amniotic epithelial cells are more likely to participate in anti-inflammation and immunoregulation, as a previous study shows that human amniotic epithelial cell transplantation improves ovarian function in POF via anti-inflammation and antiapoptosis, which is mediated by tumor necrosis factor (TNF)-α [39]. Whether, and how, BMSCs play a key role in the anti-inflammation and immunoregulation in a model of POF is still unclear. However, BMSCs play an important role in anti-inflammation and immunoregulation for other diseases, such as heart failure [40], sepsis [41], and allergic rhinitis [42]. A study suggests that BMSC paracrine activity has an anti-inflammatory effect and an antiapoptotic effect on intervertebral disc degeneration (IDD) and that this is mediated, at least in part, via the relative nuclear factor-κB (NF-κB) and mitochondrial apoptotic pathways in annulus fibrosus (AF) cells [43]. A study reports that BMSCs attenuate IL-1 by a paracrine mechanism to inhibit inflammation. The proinflammatory cytokine interferon (IFN)-γ shows a synergistic effect with BMSCs on immunosuppression, possibly by upregulating prostaglandin E2 (PGE2), HGF, and TGF-β1 in BMSCs and inducing BMSC expression of indoleamine 2,3-dioxygenase (IDO), which is involved in tryptophan catabolism [44].

Allogeneic transplantation of BMSCs is possible because of their low immunogenicity. BMSCs express low levels of major histocompatibility complex (MHC) class I molecules, and do not express MHC class II molecules which contributes to immune exemption or immune tolerance via suppressing T-cell proliferation [45]. It is reported that BMSCs have immunomodulatory effects on all types of immune cells in vitro [46, 47] and in vivo [48]. This mainly depends on the regulation of immune cells directly [49] or the balance between anti-inflammation and proinflammation by paracrine cytokines [50]; on one hand, BMSCs inhibit the function of various immune cells resulting in immune tolerance and, on the other hand, they can not only secret anti-inflammatory factors but they also suppress proinflammatory substances, thus restraining further aggravation of the “inflammatory cascade reaction” from the source. For example, BMSCs reprogram macrophages by secreting PGE2 to increase their IL-10 production [41]. BMSCs inhibit differentiation and maturation of dendritic cells by miRNA-23b [51]. BMSCs change the macrophage phenotype and inhibit local inflammation via TNF-receptor (TNF-R)2 [52]. The immunoregulatory effect is associated with HGF and TGFβ [53]. In conclusion, BMSCs may also have an important effect on anti-inflammation and immunoregulation in POF. However, the mechanism needs to be further studied.

## Oxidative stress effects of BMSCs

The disorder between free radicals and oxidative radicals, known as oxidative stress, is believed to be a potential etiology of POF [54–56]. Xiang et al. found that hPMSCs promote the recovery of ovarian function by reducing superoxide dismutase (SOD) [57]. From this, we can speculate whether BMSCs influence oxidative stress to restore ovarian function. Presently, that fact that BMSCs regulate oxidative stress to promote ovarian function in POF has not been reported, but BMSCs do have an effect on oxidative stress in other diseases, such as colitis [58].

## Problems and prospects

The transplantation of BMSCs is bringing hope for patients with POF, especially autologous BMSCs since they are not only easily obtained but also avoid graft rejection after transplantation. However, some problems still need to be resolved. Autologous BMSC transplantation may have a positive effect on patients with POF with no hematonosis. However, allogeneic BMSC transplantation can cause women with POF have to suffer graft rejection and, more seriously, they may have to endure sequelae. Clinical research demonstrates that autologous BMSC transplantation has a better therapeutic ratio (25%) than allogeneic transplantation (7%), and approximately 25% of women are more likely to face chronic gynecological graft-versus-host disease [59]. Consequently, an accurate pretreatment evaluation and frequent monitoring during treatment are required. Moreover, the counts of BMSCs and the transplantation approaches have not been optimized.

## Conclusion

Given their low immunogenicity, and the fact that they can be obtained easily and amplified in large quantities in vitro, BMSCs are a good candidate for transplantation in POF. Moreover, BMSCs migrate to the injured ovary and secret crucial cytokines that are helpful for antiapoptosis, antifibrosis, anti-inflammation, and immunoregulation which improves ovarian function. Despite the obvious effects in animal models of POF, there are some clinical problems. The therapeutic ratio of BMSC transplantation in clinical trials is not high enough to ensure that most patients with POF will recover their ovarian reserve. The molecular mechanisms of antioxidant, anti-inflammation, and immunoregulation are still to be uncovered. Furthermore, in clinical trials, the counts of BMSCs and the transplantation approaches need to be optimized so that BMSC transplantation has a higher therapeutic ratio in the clinic.

## Abbreviations

ADM: Adrenomedullin
AF: Annulus fibrosus
AMH: Anti-mullerian hormone
bax: Bcl-2-associated X protein
Bcl-2: B-cell lymphoma-2
bFGF: Basic fibroblast growth factor
BMSC: Bone marrow-derived mesenchymal stem cell
c-myc: Proto-oncogene
CXCL8: C-X-C chemokine ligand-8
Cyp19: Aromatase
ET-1: Endothelin-1
FGF-2: Fibroblast growth factor-2
FSH: Follicle-stimulating hormone
GMCSF: Granulocyte macrophage colony-stimulating factor
HGF: Hepatocyte growth factor
hPMSC: Human placenta-derived mesenchymal stem cell
IDD: Intervertebral disc degeneration
IDO: Indoleamine 2,3-dioxygenase
IFN: Interferon
IGF: Insulin-like growth factor
IL: Interleukin
LMO2: LIM-domain only 2
MCP1: Monocyte chemoattractant protein 1
MHC: Major histocompatibility complex
miR-21: MicroRNA-21
MMP: Matrix metalloproteinase
MT1: Membrane type 1
NF-κB: nuclear factor-κB
Notch: Translocation-associated
p21: Cyclin-dependent kinase inhibitor 1A
PDCD4: Programmed cell death 4
PDGF: Platelet-derived growth factor
PGE2: Prostaglandin E2
PI3K/Akt: Phosphatidylinositol-3-OH-kinase/protein kinase B
POF: Premature ovarian failure
PTEN: Phosphatase and tensin homolog deleted on chromosome ten
SOD: Superoxide dismutase
TGF: Transforming growth factor
TIMP: Tissue inhibitors of matrix metalloproteinase
TNF: Tumor necrosis factor
TNF-R: Tumor necrosis factor receptor
Treg: Regulatory T
VEGF: Vascular endothelial growth factor
